# Exsection of the Femur for Gun-shot Injury

**Published:** 1876-04

**Authors:** Conrad Deihl


ART. III.—*Exsection of Femur for gun-shot injury. Five inches of bone removed, followed by complete recovery, with only three inches shortening.* By Conrad Deihl, M. D.

When the authors of Military Surgery are consulted, we are surprised to find how generally fatal is compound, comminuted fracture of the upper third of the femur, from gun-shot injury. Amputation has almost uniformly been practiced and recommended. Erichsen, in speaking of gnn-shot injuries, says: "Severe compound and comminuted fractures of the femur, with destruction of surrounding tissue, even though the vessels and nerves be not injured, demand amputation." He gives the ratio of motality as 86 per cent.; amputation at hip-joint, 100 per cent., or *all* fatal. The following case is in all respects interesting and instructive, and is reported as showing, as well as any one case can, the advantages of true conservatism in operative surgery. Sergeant Schwinn of Buffalo, was out target-shooting, when by accident a minnie ball struck the right femur about four inches below the trochanter major, producing compound, comminuted fracture of the femur, with destruction of much tissue. He was seen by Dr. Marcley of Black Rock, soon after the accident, who dressed the wound temporarily, and had him removed to his residence in the city. Seeing the gravity of the injury, several experienced physicians were called in consultation with Dr. Marcley and myself; among the number, Prof. Julius F. Miner, who, upon arrival, and after making examination, advised Exsection instead of amputation, remarking, that amputation at the hip joint, or so near it, would be fatal. The experienced physicians present all heartily seconded the proposal, and Dr. Marcley invited Dr. Miner to conduct the operation.

The operation consisted in projecting the ends of the bone through the lacerated wound, and sawing them off as smoothly as possible. Fifty pieces, varying from $\frac{1}{4}$ to $4\frac{1}{4}$ inches in length, were removed, making in all, five inches of the shaft. Hemorrhage not being troublesome, the leg was dressed in straight position with side splints, no effort being made to approximate the soft parts. The muscles were allowed to contract, with the view of bringing the bones in apposition, and the hope of obtaining bones union, the amount of shortening being considered as comparatively unimportant. To save the life was the principal object; shortening and deformity being left for future consideration. By arrangement of Drs. Marcley and Miner, the patient was now left in my care. By means of side splints and simple dressings I was able to keep the thigh in proper place in straight position, until complete union of the wound and bone. No very unfavorable symptoms were seen after the operation. The case progressed favorably and was dismissed *well*, bony union being complete in thirteen weeks from time of accident, with about three inches shortening, or two inches less than the length of the bone removed by Exsection, this being one of the remarkable features of the case. Patient now walks comfortably upon the leg, the shortening being relieved, in part, by cork sole; so that the usual gait upon the street can scarcely be seen to be at all defective.

